# Iatrogenic aortic and iliac artery transections during robotic nephrectomies requiring emergent open vascular repair

**DOI:** 10.1016/j.jvscit.2026.102417

**Published:** 2026-07-20

**Authors:** Christopher Lightfoot, Neysa Sanghavi, Teng Teng, Mahalingam Sivakumar, Alok Aggarwal, Nelson Menezes

**Affiliations:** a Department of Surgery, The Brooklyn Hospital Center, Brooklyn, NY; b St George’s University School of Medicine, True Blue, Grenada

**Keywords:** Aortic transection, Iliac artery injury, Interposition graft, Open conversion, Robotic nephrectomy, Vascular injury

## Abstract

**Background:**

Robotic and laparoscopic radical nephrectomy are widely performed and are generally considered safe. Major vascular injuries are rare but potentially life-threatening, most often involving extrarenal visceral arteries. In contrast, injury to the aortoiliac axis during minimally invasive nephrectomy is exceptionally rare, and complete intraoperative transections have been seldom documented.

**Methods:**

We report three cases of major aortoiliac vascular injury occurring during robotic nephrectomy between 2021 and 2024. Clinical presentation, mechanism of injury, intraoperative management, vascular reconstruction, and postoperative outcomes were reviewed.

**Results:**

Two patients sustained complete infrarenal aortic transections and one sustained a right common iliac artery transection. All injuries occurred during stapled division of a misidentified renal vessel, requiring emergent conversion to open surgery with interposition graft reconstruction. Distal perfusion was restored in all cases, and the patients experienced uncomplicated postoperative recoveries without further vascular compromise.

**Conclusions:**

Life-threatening arterial injuries during robotic or laparoscopic nephrectomies commonly result from anatomic misperception of distorted planes rather than proximity to major vessels alone. Prevention requires a structured review of preoperative imaging to confirm anatomic landmarks and intraoperative verification of vessel origin and course before division. In addition, there should be a low threshold for conversion to open surgery when anatomy is uncertain. In the event of major vascular injury, rapid conversion, prompt proximal and distal vascular control, and early intraoperative collaboration with vascular surgery are critical to achieving favorable patient outcomes.

Laparoscopic and robotic radical nephrectomy are both well-established approaches for management of renal masses and are generally associated with improved perioperative recovery compared with open surgery. However, major vascular injury remains one of the most consequential intraoperative complications in minimally invasive surgery. Reviews of nephrectomy-related vascular injuries have emphasized that, although these events are uncommon, they require immediate recognition and a rehearsed escalation pathway to avoid irreversible ischemic sequelae or exsanguination.^1^

Most vascular events during left radical nephrectomy involve extrarenal visceral arteries, particularly the superior mesenteric artery (SMA), celiac axis, and contralateral renal vessels.^1^ These injuries are frequently attributed to anatomic proximity at the left renal hilum, anatomic variation, and challenging hilar dissection, especially when tissue planes are distorted by inflammation, dense adhesions, tumor-related desmoplasia, or previous interventions.^1^ Notably, the robotic surgery literature indicates that proximity alone does not fully account for severe injuries. Sayegh et al^2^ reported intraoperative SMA injury during left robotic radical nephrectomy when the SMA was misidentified as the left renal artery, underscoring anatomic variation and spatial disorientation as mechanisms of harm. This is clinically significant because a surgeon may proceed confidently on a structure presumed to be correct, particularly when local visual cues coincide with expectations.

Injury to aortic and iliac arteries during minimally invasive nephrectomy are exceptionally rare compared with visceral arterial injuries. Published reports describe partial aortic ligation or transection during laparoscopic nephrectomy, often identified by postoperative ischemic complications.^3^, ^4^, ^5^ As a result, there is a limited evidence base concerning complete intraoperative large vessel transections during robotic nephrectomy, with even fewer reports explicitly attributing such events to orientation or landmark failures in minimally invasive surgery.

We report a three-case series between 2021 and 2024 of large vessel transections during robotic nephrectomy, including two cases of complete infrarenal aortic transections and one case of right common iliac artery transection. Each of these cases resulted from vascular misperception and required emergent open conversion and interposition graft reconstruction. This case series aims to contribute to the limited literature on catastrophic arterial injuries during minimally invasive nephrectomy and to reinforce prevention and remedial strategies centered on structured anatomic confirmation, rapid conversion, and multidisciplinary vascular management. Written informed consent for publication of clinical details and images was obtained from all patients in accordance with institutional standards.

## Case 1: aortic injury

**Fig 1:**
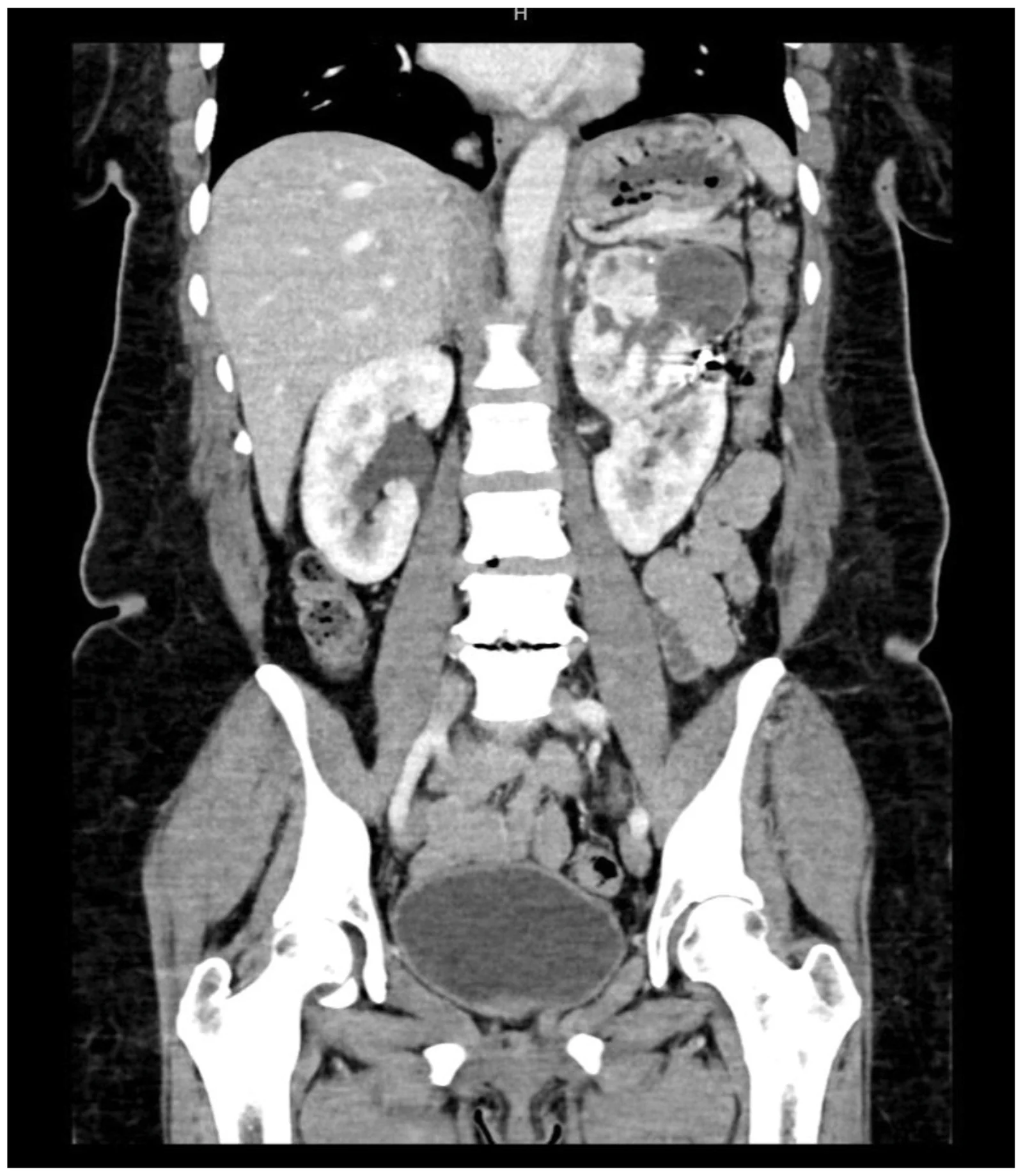
Solid, partially necrotic mass arising from the upper pole of the left kidney, measuring 8.7 × 7.1 × 8.4 cm. Suture material and embolization coil are seen within the mass. The mass compresses the upper pole calyx but is predominantly exophytic and does not impinge upon the collecting system. There is no hydronephrosis. The right kidney is unremarkable.

**Fig 2:**
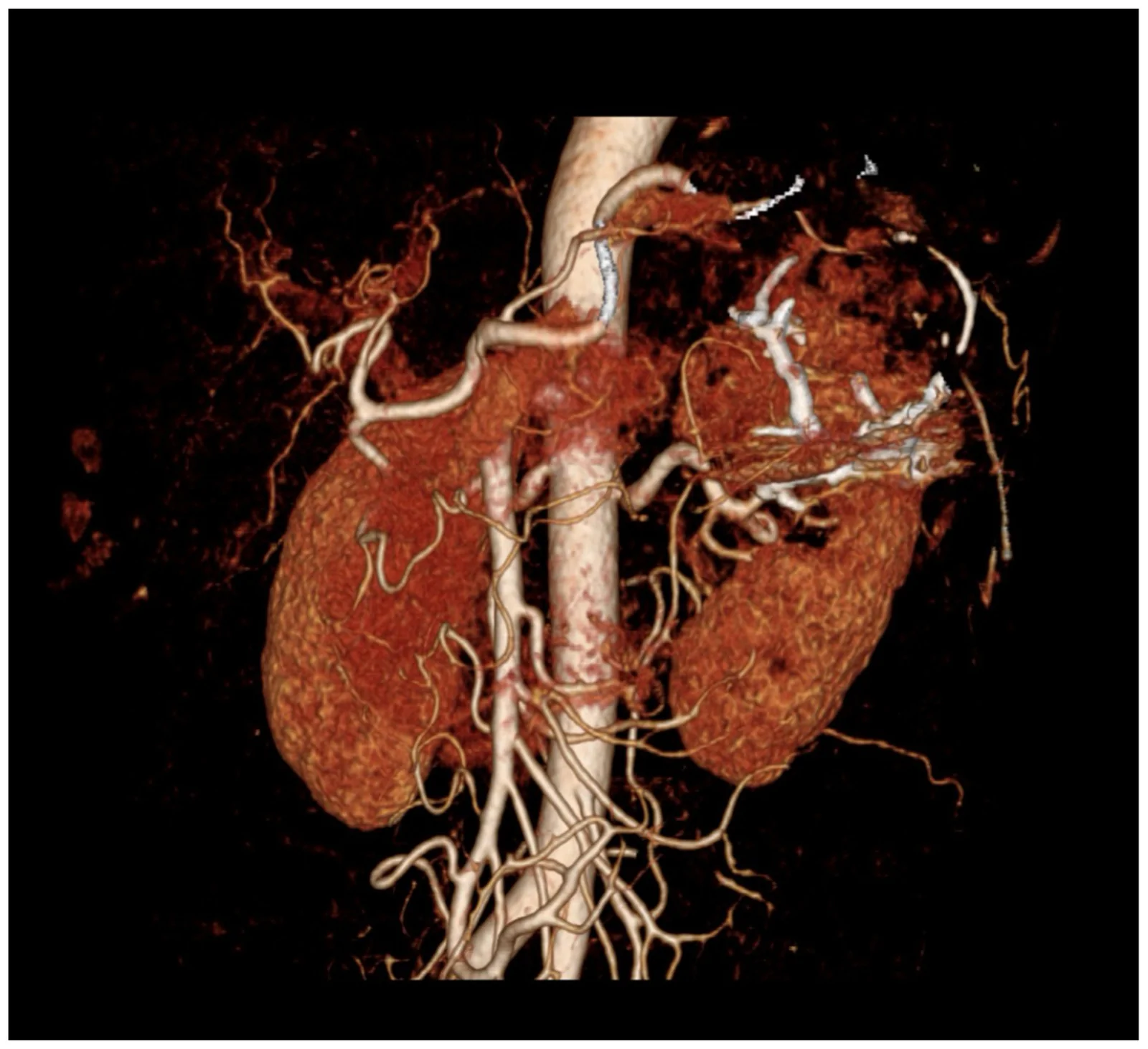
Preoperative three-dimensional reconstruction of the aorta demonstrating no aberrant vascular anatomy.

**Fig 3:**
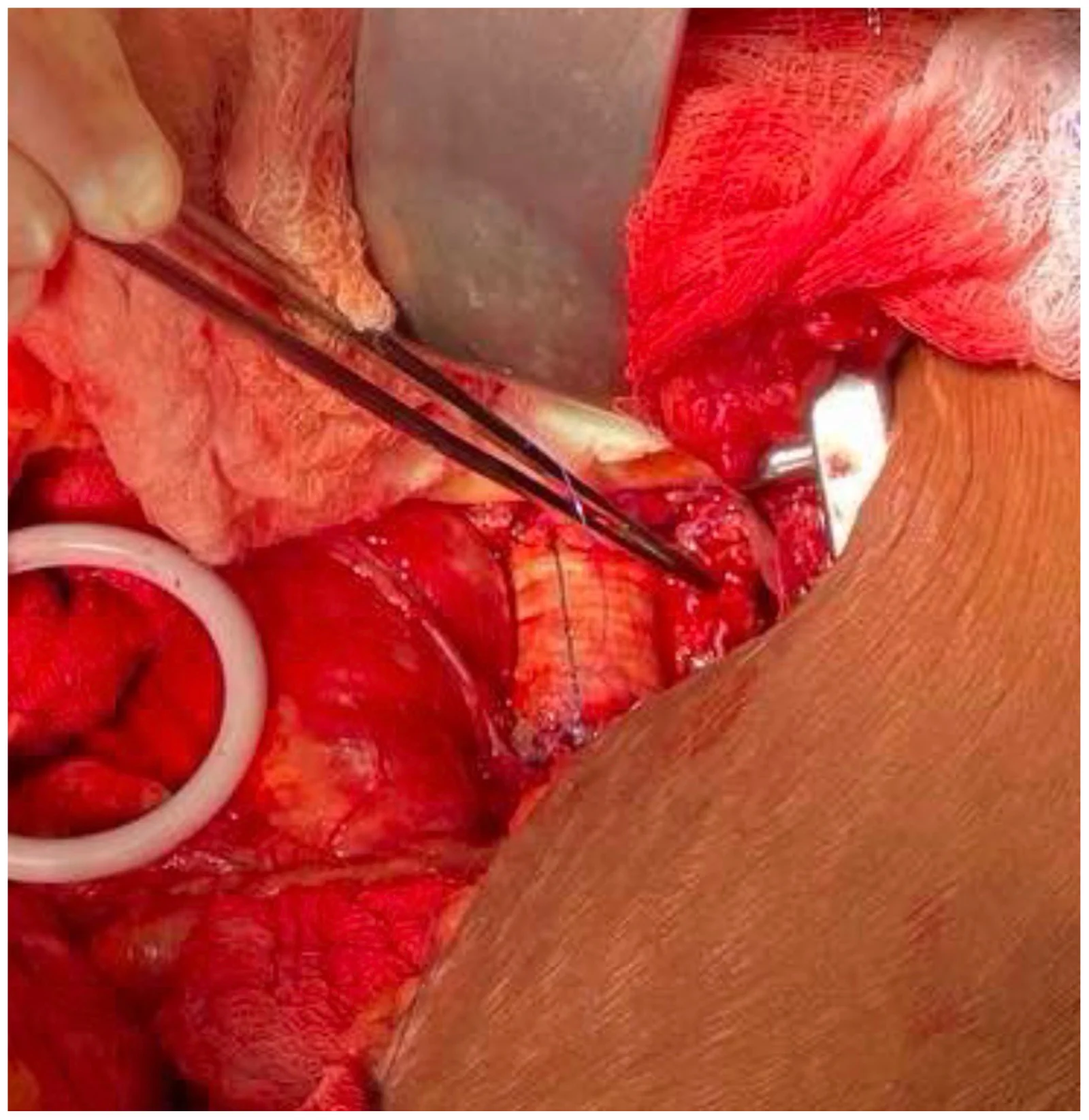
Interposition graft reconstruction of the aorta.

A 60-year-old individual was found to have a necrotic 8 cm mass in the upper pole of the left kidney, underwent robotic left total nephrectomy (Fig 1). The patient was positioned in the right lateral decubitus position at the time of injury. Preoperative imaging, including a three-dimensional reconstruction of the aorta, did not demonstrate aberrant vascular anatomy (Fig 2). Intraoperatively, dense scarring was noted over the left renal hilum likely from the mass. The upper pole of the kidney was isolated from the surrounding structures. The left renal vein was coursing over an artery which was misidentified as left renal artery. This artery was going directly into the sclerotic area with fibrosis where the left renal vein joins the hilum of kidney. After the artery was transected with robotic vascular stapler and upon division, the transected artery was correctly identified as infrarenal aorta due to its larger diameter. Vascular surgery was consulted. The patient's feet were warm, but there were no palpable pulses in the lower extremity. Monophasic signals were present over the dorsalis pedis bilaterally. Immediately, the patient underwent an exploratory laparotomy and repair of the aorta. Exploration confirmed stapled transection of infrarenal aorta at an angle in its distal one-third. The transection was distal to the renal arteries, although an exact measurement from the renal artery origins was not made. A 16 mm Hemashield tube graft (Getinge AB) was placed in an end-to-end fashion between the proximal and distal aortic stumps (Fig 3). Palpable femoral and dorsalis pedis pulses were noted bilaterally. Final pathology of the renal lesion returned as an oncocytoma with extensive necrosis. As the mass was found to be benign, her surgery represented definitive management of the lesion.

## Case 2: aortic injury

**Fig 4:**
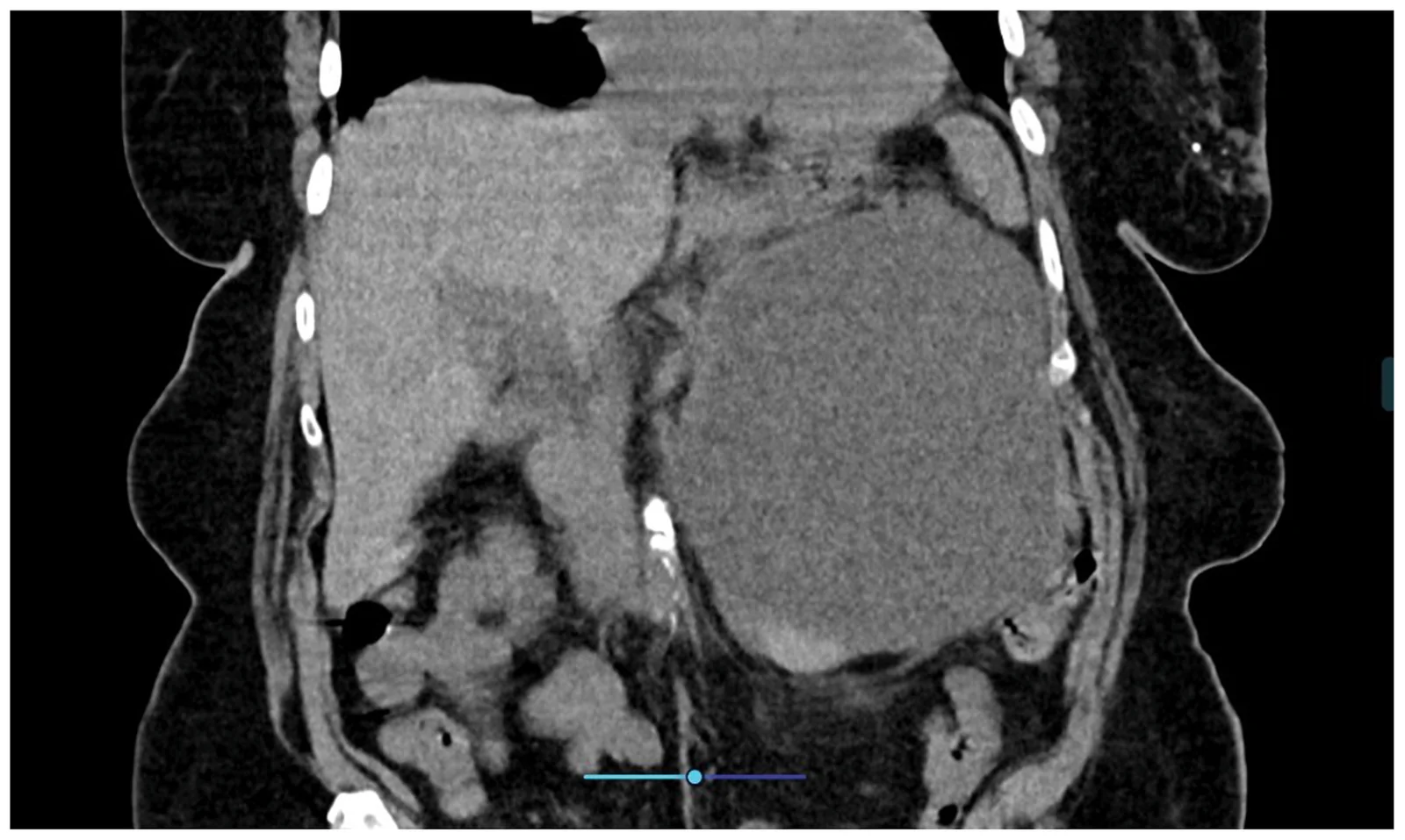
Complex left renal cystic mass measuring up to 17.9 × 12.6 × 16.6 cm, previously 17.2 × 12.6 × 15.5 cm. A hyperdense component is again noted along the posterior aspect of the lesion, corresponding to T1-hyperintense products on previous magnetic resonance imaging (MRI). No new solid component is identified, although evaluation is limited by the absence of intravenous contrast. Right renal cysts are again visualized, the largest measuring up to 3.0 cm, previously 3.4 cm. There is no noncontrast evidence of a suspicious right renal mass, renal calculus, or hydronephrosis.

**Fig 5:**
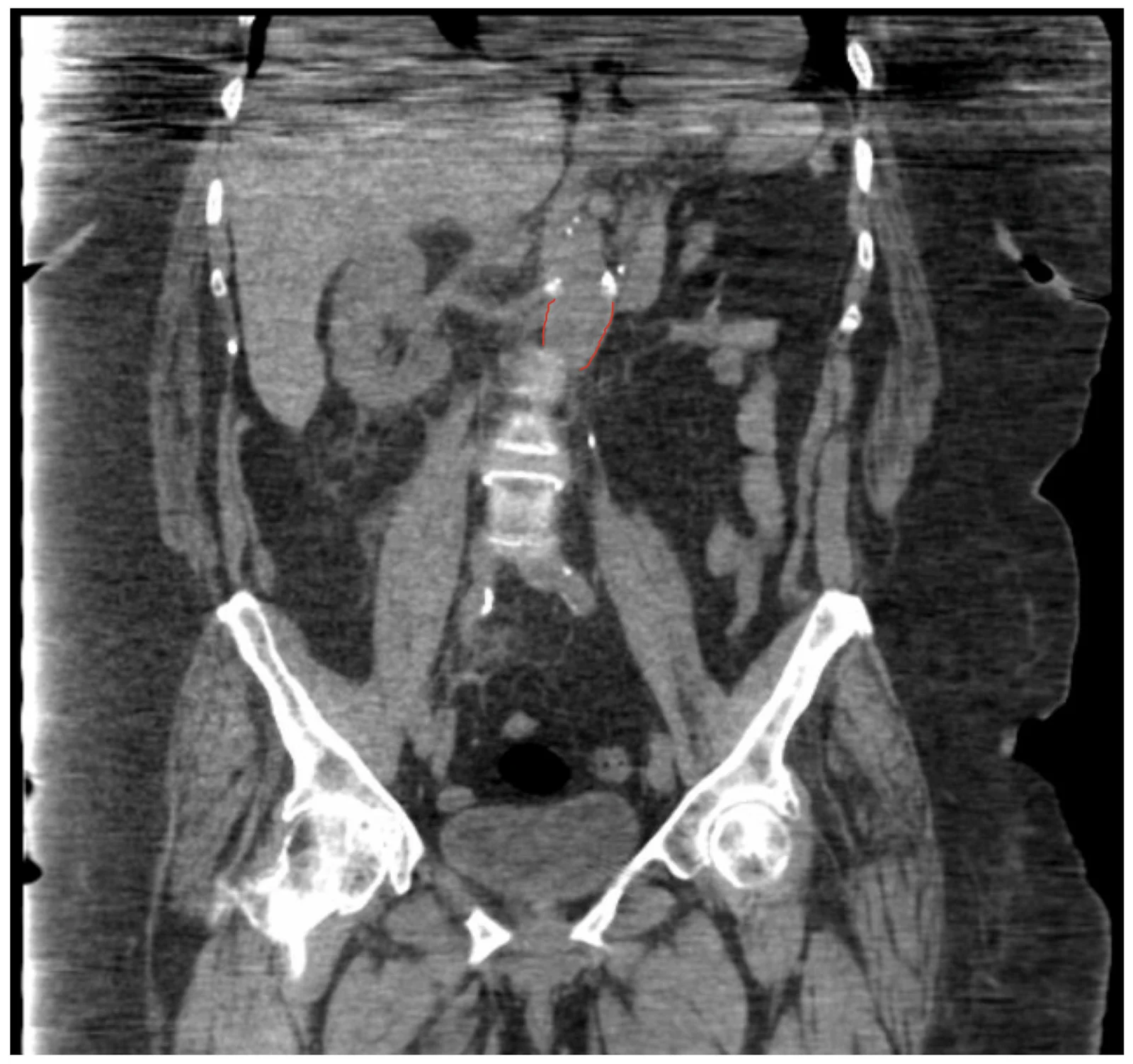
Postoperative noncontrast computed tomography (CT) demonstrating the aortic interposition graft, outlined in *red*.

A 79-year-old patient with a 17 cm left renal cyst was scheduled for robotic left radical nephrectomy (Fig 4). The patient was positioned in the right lateral decubitus position at the time of injury. Intraoperatively, visualization and working space were significantly limited by the cyst. Mobilization of the colon was challenging; therefore, cyst aspiration was performed to improve exposure. A small window was created superior to the hilum, and the renal hilum was transected with a vascular stapler. Intraoperative bleeding necessitated deployment of a second staple load, after which hemostasis was achieved. Following specimen resection approximately 2 hours into the procedure, inspection of the hilum revealed inadvertent transection of the infrarenal aorta. Vascular surgery was emergently consulted. The patient’s lower extremities were cool, with monophasic Doppler signals. Exploration confirmed stapled transection of the infrarenal aorta. The injury was just distal to the renal artery origins. An 18 mm Hemashield tube graft was placed in an end-to-end fashion between the proximal and distal aortic stumps. Before completion of the distal anastomosis, a 5F embolectomy catheter was passed into the iliac arteries, demonstrating satisfactory back-bleeding without thrombus. The inferior mesenteric artery was identified and preserved. After full restoration of aortic flow, strong bilateral femoral pulses were confirmed. Because of the patient’s significant baseline renal dysfunction, no preoperative or postoperative contrast-enhanced imaging was obtained. A postoperative noncontrast computed tomography (CT) has been added to demonstrate the graft (Fig 5). Gross pathology demonstrated a 14 × 13 × 6 cm specimen with perinephric fat. The renal parenchyma was largely replaced by a 9.5 × 9 cm cystic lesion, with a 4.8 × 3.0 cm solid upper pole mass. Final pathology revealed a low-grade oncocytic renal tumor.

## Case 3: iliac injury

**Fig 6:**
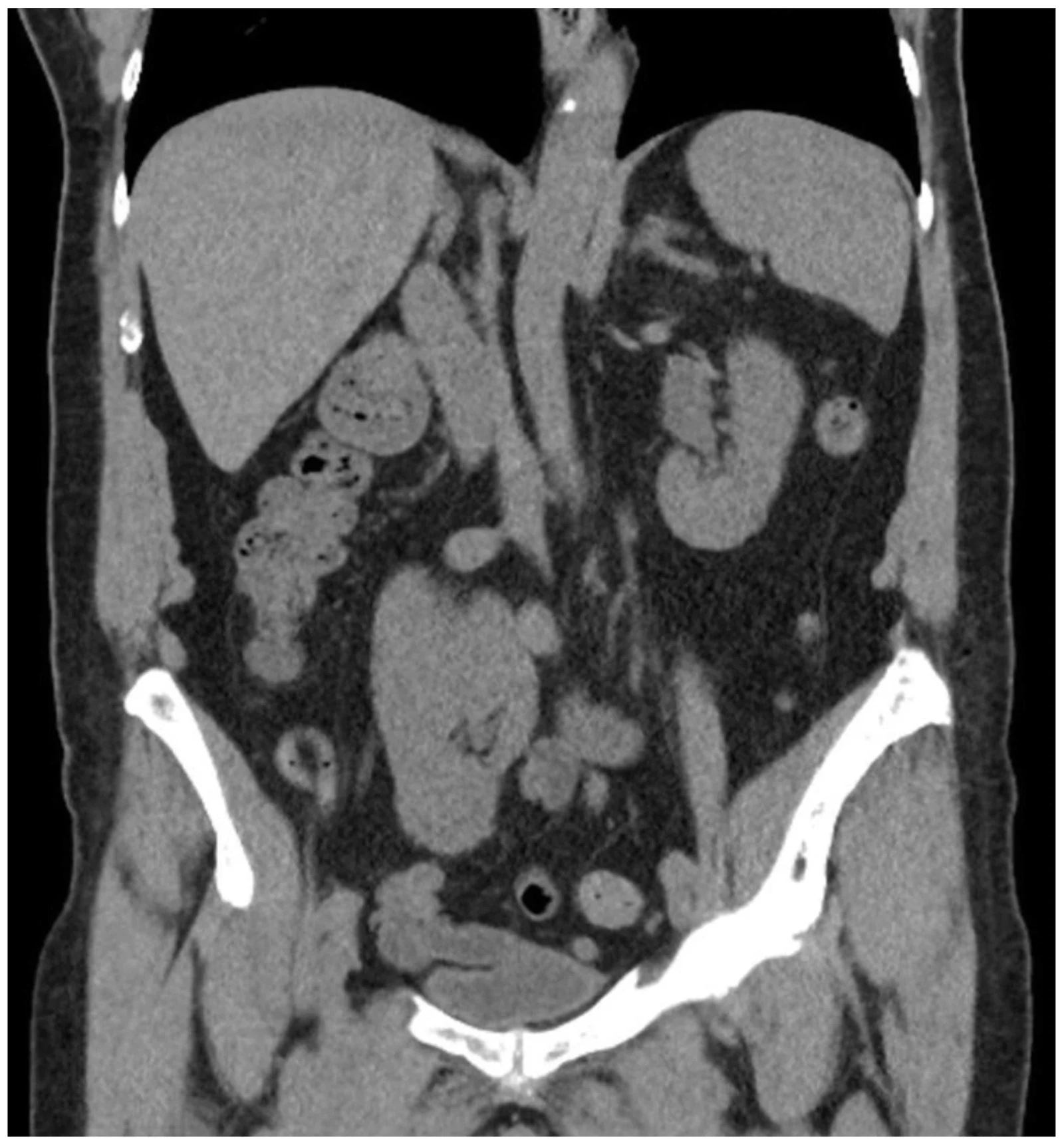
Ptotic positioning of the malrotated right kidney in the right lower abdomen. A solid renal mass is seen in the lower pole of the right kidney, measuring 4.1 × 3.9 cm.

**Fig 7:**
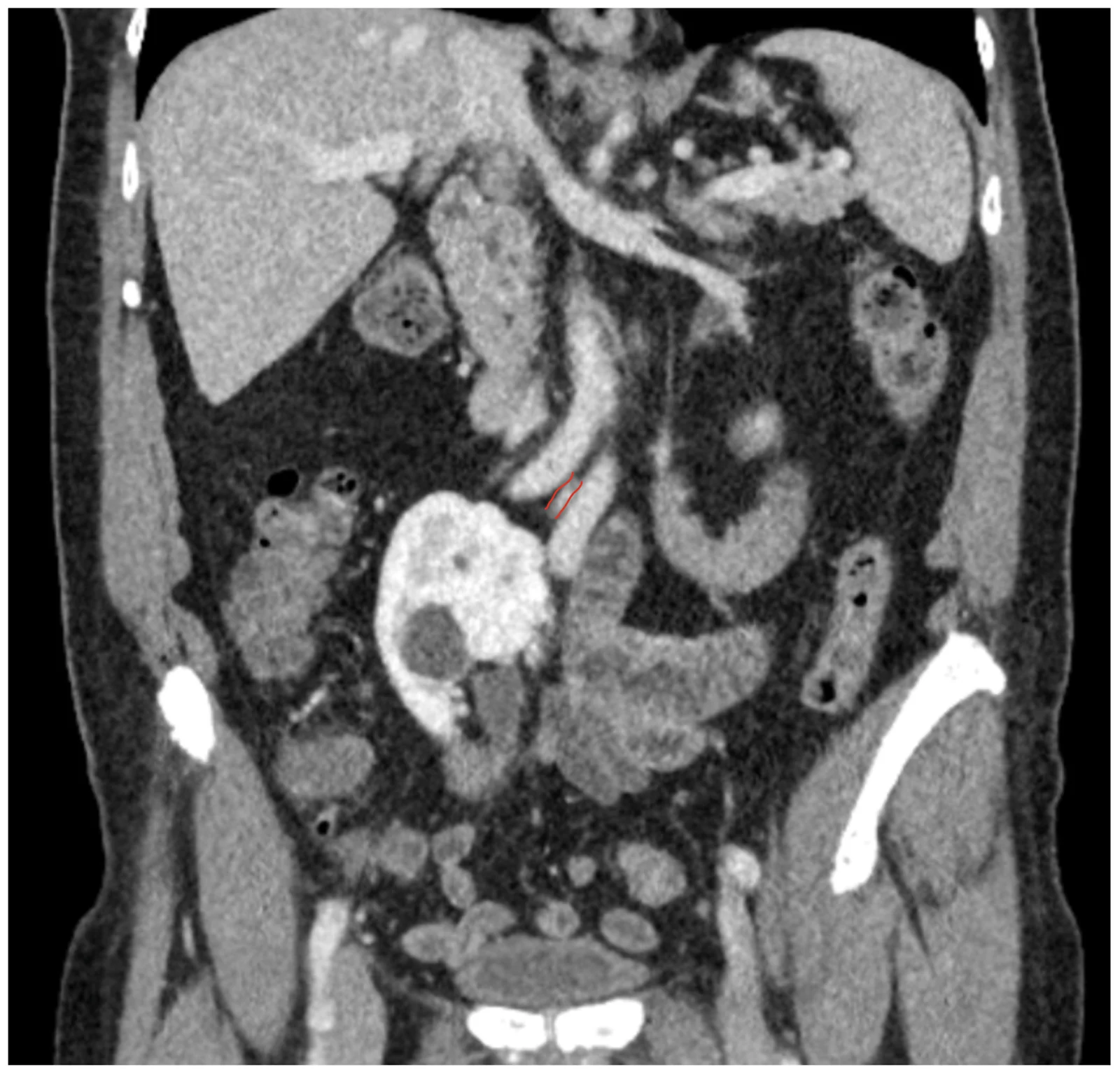
Contrast-enhanced computed tomography (CT) urogram slices with the right renal artery highlighted in *red*, demonstrating the relationship of the low-lying malrotated right pelvic kidney to the adjacent right iliac artery.

A 78-year-old man found to have a right lower pole renal mass of 4.1 × 3.9 cm in diameter consistent with renal cell carcinoma papillary grade 2, underwent robotic right nephrectomy. Preoperative scans documented low-lying, malrotated right pelvic kidney (Fig 6). Additional contrast-enhanced CT urogram slices demonstrate the right renal artery and its relationship to the low-lying malrotated kidney and adjacent right iliac artery (Fig 7). The patient was positioned in the left lateral decubitus position at the time of injury. Intraoperatively, there was significant desmoplastic reaction and scarring noted around the entire kidney and hilum. The kidney was clearly attached to the psoas and the vena cava. Sharp dissection of scarred tissue was performed thus circumferentially dissecting the kidney and identifying the presumed renal artery at the hilum which was staple-divided. Upon transection, there was suspicion of common iliac artery transection due to its stapled end proximity to patient’s high aortic bifurcation. Vascular surgery was emergently consulted intraoperatively, and right distal lower-extremity pulses were nonpalpable. Exploratory laparotomy confirmed stapled transection of right common iliac artery. An interposition 8 mm polytetrafluoroethylene graft was placed in an end-to-end fashion between the proximal and distal iliac stumps. Full restoration of iliac flow with strong distal pulses were confirmed on Doppler.

## Discussion

Minimally invasive radical nephrectomy is widely implemented due to its faster patient recovery, durable oncologic outcomes, and reduced perioperative morbidity compared with open surgical approaches.^6^ Despite this favorable safety profile, major vascular injury remains one of the most significant intraoperative complications in laparoscopic and robotic procedures.^7^, ^8^, ^9^ Bleeding and distorted anatomy can rapidly impair perception. Large series and pooled analyses consistently demonstrate low overall rates of major complications.^1^^,^^10^ However, these studies also emphasize that vascular injury, although infrequent, remains among the most consequential intraoperative events because it can rapidly result in exsanguination, end-organ ischemia, and possible increase in overall morbidity and mortality. The presented case series describe major arterial transections during robotic nephrectomy; two involving infrarenal aorta and one involving right common iliac artery. In each instance, injury resulted during stapled division of a presumed renal vessel and was recognized intraoperatively followed by emergent conversion to open surgery. This enabled immediate vascular control and reconstruction which reduced any delay in recognition and treatment resulting in more favorable prognosis.

Published literature reports partial aortic injury or postoperative identification of aortic compromise rather than complete intraoperative transection.^3^^,^^4^ Fei et al^3^ described partial supraceliac aortic transection from stapling during laparoscopic nephrectomy that presented with acute limb and mesenteric ischemia which required resection with thoracoabdominal bypass plus a celiac jump graft. Almudaiheem et al^4^ reported delayed complete infrarenal aortic occlusion after right robotic nephrectomy due to Hem-o-lok clips (Teleflex Incorporated) clamping the aorta, diagnosed on CT angiography and treated with prosthetic interposition grafting. Reports of iliac artery injury during nephrectomy are equally rare, though it represents a recognized complication in pelvic and retroperitoneal laparoscopic surgery.^5^^,^^11^^,^^12^ Bertolo et al^5^ described a case of right external iliac artery injury in nephroureterectomy where endometriosis, a benign infiltrative disease, distorted planes and increased the risk of iliac vascular injury.

Understanding the pattern of major arterial injury during nephrectomy is vital for interpreting mechanisms and developing preventive approaches. In this case, the mechanism is best explained by anatomic misperception in a hostile hilum rather than by proximity-based injury. The literature on life-threatening arterial injuries during nephrectomy most frequently describes injuries to extrarenal visceral arteries, particularly the SMA and celiac axis, often when these vessels are mistaken for the left renal artery during challenging hilar dissection.^1^, ^2^, ^3^, ^4^^,^^13^ Limited academic literature and narrative reviews emphasize that vascular injuries commonly stem from the surgeon committing to a vessel that appears correct before its origin and course are conclusively established.^1^, ^2^, ^3^, ^4^

Our operative findings mirror these reports, in which a vessel presumed to be the renal artery was stapled, and the error became evident only after proximal filling was observed, raising suspicion of aortic or iliac transection that was confirmed intraoperatively by absent distal pulses in the lower extremities. Contributing factors included hostile hilar anatomy, previous embolization procedures, scarring, high bifurcation of aorta with malrotated pelvic kidney, and loss of normal retroperitoneal planes. In hostile hilar anatomy, preventing catastrophic vascular injury requires deliberate, structured anatomic confirmation before any irreversible step. It is critical to re-establish fixed landmarks, reidentify the aorta as a midline reference, and tracing any presumed renal artery to a confirmed renal takeoff. Safe stapling practice by visualizing the distal ends of the tips would further prevent collateral injuries to nearby vessels. In addition, stop points can be included during the minimally invasive surgery where the team stops to confirm (1) the psoas and gonadal vein/ureter relationship, (2) the renal vein course into the inferior vena cava, (3) the expected takeoff and direction of the renal artery relative to the vein, (4) the absence of too-large or too-midline caliber or pulsatility inconsistent with a renal artery, (5) and possible test-clamping the vessel before division.^12^^,^^14^

If these checks cannot be satisfied, early conversion should be treated as a deliberate safety step to regain exposure and anatomic certainty. With major vascular injury, outcomes depend on rapid recognition, prompt conversion, early vascular surgery involvement, and swift proximal/distal control. Across all three patients in this series, immediate conversion and interposition graft reconstruction resulted in restoration of distal pulses and favorable outcomes. Because of the severity of the crush injury to the affected segments of aorta and common iliac caused by the staple loads, endovascular approaches were untenable. The proximal and distal edges of the transected vessels required debridement in order to reach healthy enough tissue for repair. Interposition grafting was the best option for the patients involved and all patients had positive outcomes due to the method used.

## Funding

None.

## Disclosures

None.
